# Exploring the role of milk yield in the first week of lactation on the association between hyperketonemia and reproductive performance in dairy cattle

**DOI:** 10.3168/jdsc.2021-0129

**Published:** 2021-11-25

**Authors:** Z. Rodriguez, E. Wynands, E. Shepley, L.H. Baumgard, G. Cramer, L.S. Caixeta

**Affiliations:** 1 Department of Veterinary Population Medicine, University of Minnesota, St. Paul 55108; 2 Department of Animal Science, Iowa State University, Ames 50011

## Abstract

The objective of this retrospective cohort study was to investigate whether the association of hyperketonemia (HYK) with reproductive performance differs based on milk production during the first week of lactation (wk 1). Data between calving and 150 d in milk (DIM) from 2,091 Holstein dairy cows from 5 commercial dairy herds in Minnesota were collected. The concentration of β-hydroxybutyrate (BHB) was measured twice between 3 and 10 DIM in whole blood to diagnose HYK (defined as BHB ≥1.2 mmol/L). The average wk 1 milk yield was classified into 3 levels (low = lower 25th percentile; mid = 25th to 75th percentile; high = upper 75th percentile) according to parity and herd. Log-binomial regression, Cox-proportional hazard regression, and linear regression models were used to investigate the association of HYK with pregnancy to first insemination, pregnancy by 150 DIM, and calving-to-conception interval (CCI) between groups, respectively. To describe the differences in these estimates between each wk 1 milk yield level, an interaction term of HYK and stratified levels of wk 1 milk yield was added. The incidence of HYK between 3 to 10 DIM was 14.2% (4.6% in primiparous cows and 19.4% in multiparous cows). In the study population, the effect of HYK on reproduction was greater in cows with low milk yield. Among cows with low milk yield levels, HYK was associated with lower risk of pregnancy to first insemination [risk ratio = 0.62; 95% confidence interval (CI): 0.39–0.98], lower risk of pregnancy up to 150 DIM (hazard ratio = 0.70; 95% CI: 0.51–0.96), and extended days of CCI (mean difference = 13.0; 95% CI: 5.1–20.9). In contrast, among cows with mid and high wk 1 milk yield levels, hyperketonemic (HYK+) cows had a similar reproductive performance to non-HYK (HYK−) cows. Our results suggest that early-lactation milk yield plays a role in the association of hyperketonemia with reproductive performance. These results merit further investigation to determine the role that early milk yield has in the association of hyperketonemia with health and productivity outcomes.

## Highlights

• No clear evidence was found to support an association between hyperketonemia and reproductive performance.
• Hyperketonemia was associated with lower risk of pregnancy among low-yielding cows.
• Early lactation plays a role in the association of hyperketonemia with reproduction.

## Graphical Abstract

**Figure:**
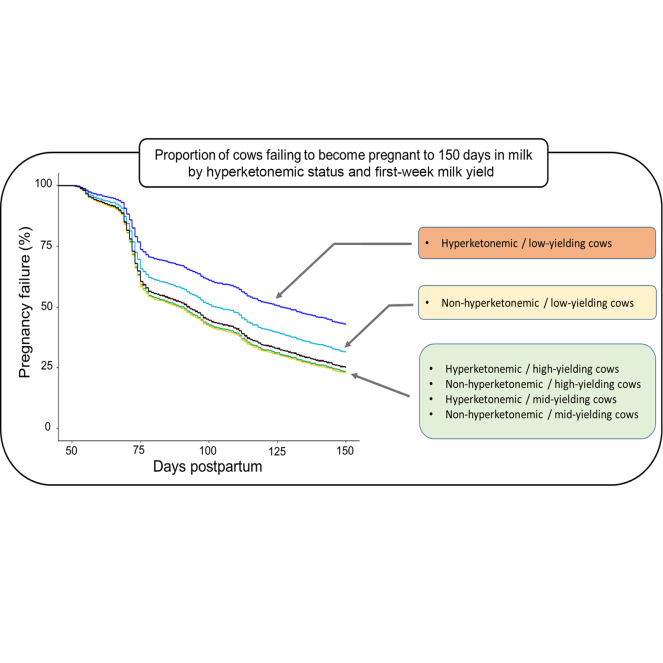
**Summary:** Hyperketonemia (HYK) is a metabolic disorder in dairy cattle related to difficulties during the transition period and associated with suboptimal reproductive performance. Studies have proposed the monitoring of the transition period based on early-lactation milk yield. Therefore, our objective was to explore the role of milk yield during the first week of lactation on the relationship between HYK and reproductive performance. Cows (N = 2,091) were tested for HYK (blood BHB ≥1.2 mmol/L), between 3 and 10 DIM. First-week milk yield was classified into 3 levels according to parity and herd. Reproductive performance was assessed until 150 DIM. After adjustments, we found no evidence to support an association between HYK and reproduction. However, models stratified by early milk yield showed that HYK was associated with lower risk of pregnancy among low-yielding cows. Results suggests that early milk yield plays a role in the association of hyperketonemia with reproduction.

The increase in the blood concentration of circulating ketone bodies during early lactation is an indication that the cow is utilizing body reserves to fulfill the energy requirement of her new physiological state (Herdt, 2000). However, an excessive presence of ketone bodies jeopardizes health and productivity. Some studies have shown that elevated concentrations of circulating BHB, also known as hyperketonemia (**HYK**), are associated with an impaired reproductive performance (Walsh et al., 2007; Ospina et al., 2010; Rutherford et al., 2016).

The evaluation of milk production during early lactation is a readily available, objective, and standardized source of information that can be used as a screening tool to monitor transition cow performance and detect individual cows that are more likely to have experienced problems during the transition period (Nordlund and Cook, 2004). In addition, monitoring of the transition period based on early lactation performance (e.g., daily milk yield, average milk yield in the first 7 DIM, and the difference between actual and expected milk yield) has been proposed as a useful predictor of the probability of cows remaining healthy in early lactation and of the probability of culling (Lukas et al., 2015).

This study was designed to explore the role of milk yield during the first week after parturition on the relationship between HYK and reproductive performance. Given that not all cows with HYK suffer negative outcomes, wk 1 milk yield might be of help in identifying cows at a higher risk of negative health and productive outcomes, allowing for further targeted interventions. The study was designed to be exploratory in nature to generate hypotheses to be tested in future confirmatory studies.

The University of Minnesota Institutional Animal Care and Use Committee approved all procedures performed during this study (protocol # 1603–33595A). This study used data originally collected to evaluate the relationship between HYK and hoof lesions (Wynands and Cramer, 2018). The data were collected from 5 commercial dairy herds in Minnesota from January to December 2017. All cows that calved during the collection period (n = 2,091) were enrolled independently of parity. Blood BHB concentration was measured twice between 3 and 10 DIM. Blood samples were collected from the coccygeal vessels when cows returned to their pen after morning milking. Immediately after collection, blood BHB concentrations were measured using an electronic handheld device (NovaVet; Nova Biomedical Co.), which was previously validated for use in dairy cows (Bach et al., 2016; Rodriguez et al., 2021). A calibration slope of 1.0 (default setting) was used to adjust for differences in hematocrit and to maximize the sensitivity and specificity of the test (Rodriguez et al., 2021). Calving information, health events, culling, monthly DHI records, and reproductive events until 150 DIM were entered in the farm management software (DC305, Valley Ag Software) by farm personnel.

In 4 of 5 herds, cows received their first insemination by timed AI following a double-Ovsynch fertility program (GnRH–7 d–PGF_2α_–3 d–GnRH–7 d–GnRH–7 d–PGF_2α_–56 h–GnRH–17 ± 1 h–AI). For these herds, the voluntary waiting period varied between 65 ± 3 and 70 ± 3 d. In the remaining herd, cows were pre-synchronized (PGF_2α_–14 d–PGF_2α_) 12 d before the initiation of an Ovsynch protocol (GnRH–7 d–PGF_2α_–56 h–GnRH–17 ± 1 h–AI). Cows were inseminated if detected in estrus after the second PGF_2α_ treatment of the Presynch part of the fertility program (50 ± 3 DIM). Cows that did not show estrus activity following the Presynch portion of the protocol received timed AI at 72 ± 3 DIM at the completion of the breeding Ovsynch portion of the protocol. In all herds, subsequent inseminations happened following estrus detection, and cows that did not demonstrate estrus behavior before pregnancy diagnosis examination were submitted to a Resynch protocol (GnRH–7 d–PGF_2α_–56 h–GnRH–17 ± 1 h–AI). Pregnancy diagnosis was performed between 32 ± 3 and 42 ± 3 d after breeding and reconfirmed between 60 ± 3 and 90 ± 3 d after breeding depending on the herd.

The average wk 1 milk yield was obtained in 3 herds by individual milk weights stored in the farm management software. The wk 1 milk yield reported in these farms was based on the average of all available daily milk weights. In 2 other herds, where individual daily measurements were not available, milk yield in the first week postpartum was estimated using the DC305 software equation that uses the peak milk production and preceding DHIA milk test results to predict milk yield in the first week postpartum. All information regarding milk yield in the first week postpartum was exported from DC305 using the command “W1MK.”

All statistical analyses were performed using R software, version 3.4.4 (RStudio Inc.; https://www.r-project.org/), and the individual cow was the unit of interest. The exposure variable of interest was HYK diagnosed by measuring BHB in whole blood twice between 3 and 10 DIM, 3 to 4 d apart. Cows with a BHB measurement of ≥1.2 mmol/L in at least 1 of the 2 samples taken were deemed hyperketonemia-positive (HYK+) and were otherwise hyperketonemia-negative (HYK−). Average wk 1 milk yield was categorized in 3 levels based on percentiles. The bottom 25th percentile was considered low-yielding cows, cows between the 25th to 75th percentile were grouped in a mid-yielding group, and cows in the 75th percentile were considered high-yielding cows. Percentiles were calculated separately for different parity groups (primiparous and multiparous) and herds (Table 1).

**Table 1:** Descriptive characteristics of the Holstein dairy cows included in the analyses according to herds

Descriptive characteristics	Herd A (n^1^ = 111)	Herd B (n = 579)	Herd C (n = 548)	Herd D (n = 275)	Herd E (n = 511)
Herd size^2^	700	1,300	2,050	1,300	650
Parity, n (%)					
Primiparous	43 (38.7)	182 (31.4)	182 (33.2)	122 (44.4)	192 (37.6)
Multiparous	68 (61.3)	397 (68.6)	366 (66.8)	153 (55.6)	319 (62.4)
Average milk yield,^3^ kg (SD)					
Primiparous 305ME^4^	15,000 (2,110)	13,000 (2,560)	13,100 (2,010)	13,100 (1,760)	13,400 (2,410)
Multiparous 305ME	14,600 (3,490)	13,700 (2,620)	13,900 (1,900)	12,800 (2,140)	13,900 (2,200)
Percentiles wk 1 milk yield,^5^ kg					
Primiparous					
25th	26.1	23.1	24.5	23.1	26.3
75th	33.1	34.8	32.2	29.3	33.6
Multiparous					
25th	35.2	36.3	37.6	34.9	39.5
75th	49.4	51.7	46.7	44.9	50.8
Health and reproduction parameters, n (%)					
HYK incidence	5 (4.4)	126 (21.5)	37 (6.7)	42 (15.2)	79 (15.3)
Pregnant to first insemination	45 (39.5)	271 (46.2)	182 (33.2)	132 (47.8)	180 (35.0)
Pregnant by 150 DIM	75 (65.8)	433 (73.8)	408 (74.3)	210 (76.1)	330 (64.1)

1 Number of animals enrolled in each herd.

2 Average herd size during the study period.

3 Average milk yield was estimated in A and B, and directly measured in C, D, and E.

4 305-d mature-equivalent milk production.

5 Percentiles utilized for the categorization of average first-week milk yield.

To determine the association between HYK with pregnancy to the first insemination, we performed a multivariable log-binomial regression model (package “lme4”; Bates et al., 2020). The association between HYK and pregnancy by 150 DIM was calculated using multivariable Cox proportional hazard model (package “survival”; Therneau, 2020). Individual time at risk for pregnancy was calculated as the number of days from the end of the waiting period until the date of pregnancy or right-censoring (i.e., death, loss to follow-up, or administrative censoring at 150 DIM). The association between HYK and calving-to-conception interval (**CCI**) was calculated using a multivariable linear regression model (package “lme4”). The insemination date associated with the first positive pregnancy diagnosis determined by the farm veterinarian between 32 ± 3 and 42 ± 3 d after breeding was used to estimate the CCI. Information regarding pregnancy losses that occurred between pregnancy diagnosis and confirmation of pregnancy was not captured by the report created by the farm management software and therefore not analyzed.

These associations between HYK and reproductive efficiency across each level of average wk 1 milk yield were evaluated separately by simultaneously introducing HYK, wk 1 milk yield, and their interaction (multiplicative) term (HYK × wk 1 milk yield) into the regression models described previously. To account for confounding effects, overall models (ignoring interaction) and stratified models (including an interaction term) were subjected to testing for potential confounding variables. Covariates offered to the models included calving difficulty (1 = no assistance, 2 = mild assistance, 3 = assisted by 2 or more people, 4 = mechanical traction or surgical procedure) and disease detected by farm personnel during the first 7 d of lactation (i.e., clinical mastitis, retained placenta, metritis, and displaced abomasum). In the overall models (ignoring the interaction term), wk 1 milk yield was also offered as a potential confounding variable. The confounding effects were assessed based on a 10% change in the main exposure estimate (i.e., HYK; Greenland and Pearce, 2015) using a manual backward stepwise elimination procedure. Results are discussed in the context of precision of the estimate using the confidence interval rather than statistical significance (Poole, 2001).

Our analyses included a total of 2,024 Holstein dairy cows. A total of 67 cows were excluded from the initial data set (n = 2,091) because of incomplete observations of the exposure (i.e., measurement outside the sampling range or only one measurement). We performed a sensitivity analysis to assess the effect of selecting only the 3 farms with individual milk measurements over including all 5 farms. The analyses showed similar estimates for pregnancy to the first insemination [risk ratio (**RR**) = 0.81, 95% CI: 0.56, 1.08], pregnancy by 150 DIM [hazard ratio (**HR**) = 0.77, 95% CI: 0.63, 0.95], and days of CCI (mean difference = 10.5, 95% CI: 3.49, 16.5), when using only 3 farms, indicating that the trends remained constant regardless of wk 1 milk yield determination method.

Table 1 contains herd characteristics information, wk 1 milk yield levels, and variables used in the analyses, sorted by herd. The incidence of HYK varied across herds, ranging from 4.4 to 21.5%. The overall incidence of HYK was 14.2% (289/2,024), with 4.5% (33/721) of primiparous and 19.6% (256/1,303) of multiparous cows classified as HYK+. During the first week of lactation, retained placenta was diagnosed in 6.7% of the cows, followed by metritis in 1.5%, displaced abomasum in 1.2%, and clinical mastitis in 0.9%. The average time to first insemination ranged between 60 and 71 d from calving, and the median time to pregnancy was 104 d across herds.

Based on models without wk 1 milk yield stratification, we observed a lack of evidence that HYK+ cows had a lower risk of pregnancy to the first insemination (RR = 0.86, 95% CI: 0.70, 1.06), but HYK was associated with a decrease in the risk of becoming pregnant by 150 DIM (HR = 0.83, 95% CI: 0.71, 0.97). Moreover, the length of CCI was longer for HYK+ than for HYK− cows (mean difference = 7.7, 95% CI: 3.3, 12.2). When evaluating the association between HYK and reproductive performance parameters within each wk 1 milk yield stratum, low-yielding cows presented a negative association (Table 2). Within the group of low-yielding cows, the HYK+ cows had a 38% lower risk of pregnancy to first insemination (RR = 0.62, 95% CI: 0.39, 0.98), were 30% less likely to become pregnant by 150 DIM (HR = 0.70, 95% CI: 0.51, 0.96), and had an additional 13 d of CCI (95% CI: 5.12, 20.9), compared with HYK− cows. We observed no evidence of an association between HYK status and reproductive performance parameters among mid-yielding and high-yielding cows (Table 2).

**Table 2:** Model estimates of the association of hyperketonemia (HYK) between 3 and 10 DIM with pregnancy to first insemination, pregnancy by 150 DIM, and calving-to-conception interval, with and without stratification by the first week of lactation milk yield in 2,024 Holstein dairy cows

Events	Estimates (95% CI)			
	Overall^1^	Low milk yield^2^	Mid milk yield^2^	High milk yield^2^
Pregnancy to first AI^3^	0.86 (0.70, 1.06)	0.62 (0.39, 0.98)	0.96 (0.73, 1.28)	0.93 (0.64, 1.35)
Pregnancy by 150 DIM^4^	0.83 (0.71, 0.97)	0.70 (0.51, 0.96)	0.90 (0.72, 1.13)	0.87 (0.64, 1.17)
Calving-to-conception interval^5^	7.77 (3.34, 12.2)	13.03 (5.12, 20.9)	3.36 (−3.35, 10.10)	4.68 (−4.06, 13.40)

1 Comparison between HYK-positive and HYK-negative cows without stratification by first-week postpartum milk yield average. HYK-negative cows are the reference group within each group. Cows were classified as HYK positive if they had at least one test above the cut-point (BHB â‰¥1.2 mmol/L) and were otherwise considered HYK negative.

2 Categories were calculated separately for primiparous and multiparous cows within each herd. Cows were considered low-yielding when their first-week postpartum milk yield average was under the 25th percentile; cows between the 25th and 75th percentiles were considered mid-yielding; cows over the 75th percentile were considered high-yielding.

3 Pregnancy to first insemination. Risk ratios were adjusted by disease in the first week.

4 Estimates of pregnancy by 150 DIM are reported as hazard ratios.

5 Calving-to-conception interval is reported as the mean difference in days.

The proportion of cows pregnant by 150 DIM, respectively, was 58.5% and 68.0% for HYK+ and HYK− low-yielding cows; 74.3% and 77.8% for HYK+ and HYK− mid-yielding cows; and 73.2% and 77.8% for HYK+ and HYK− high-yielding cows. Compared with HYK− high-yielding cows, HYK+ low-yielding cows were 44% (HR = 0.56, 95% CI: 0.37, 0.83) less likely to become pregnant by 150 DIM and had an additional 21.6 d of CCI (mean difference = 11.46, 95% CI: 11.46, 31.86; Table 3). The covariates that remained in the final models are included in the footnotes of Table 2.

**Table 3:** Comparison of the estimated association of hyperketonemia (HYK) status with pregnancy to first insemination, pregnancy by 150 DIM, and calving-to-conception interval, between each level of milk yield in the first week of lactation in 2,024 Holstein dairy cows

Variable	HYK positive^1^		HYK negative	
	Days to pregnancy^2^	Estimate (95% CI)	Days to pregnancy	Estimate (95% CI)
Pregnancy to first AI^3^				
Milk = high^4^	—	0.93 (0.57, 1.51)	—	Referent
Milk = mid	—	0.96 (0.65, 1.42)	—	0.99 (0.80, 1.23)
Milk = low	—	0.55 (0.30, 1.00)	—	0.89 (0.68, 1.16)
Pregnancy by 150 DIM^5^				
Milk = high	—	0.86 (0.59, 1.27)	—	Referent
Milk = mid	—	0.90 (0.66, 1.24)	—	1.00 (0.84, 1.19)
Milk = low	—	0.56 (0.37, 0.83)	—	0.79 (0.64, 0.99)
Calving-to-conception interval (d)^6^				
Milk = high	106	4.68 (−6.67, 16.04)	101	Referent
Milk = mid	105	3.63 (−5.67, 12.85)	102	0.27 (−4.97, 5.51)
Milk = low	123	21.66 (11.46, 31.86)	110	8.63 (2.36, 14.90)

1 Cows were classified as HYK positive if they had at least one test above the cut-point (BHB â‰¥1.2 mmol/L) and were otherwise considered HYK negative.

2 Days to pregnancy as estimated marginal means.

3 Estimates of pregnancy to first insemination are reported as risk ratios and adjusted by disease in the first week.

4 Categories were calculated separately for primiparous and multiparous cows within each herd. Cows were considered low-yielding when their first-week postpartum milk yield average was under the 25th percentile; cows between the 25th and 75th percentiles were considered mid-yielding; cows over the 75th percentile were considered high-yielding.

5 Estimates of pregnancy by 150 DIM are reported as hazard ratios.

6 Calving-to-conception interval is reported as the mean difference in days.

Multiple studies have evaluated milk production as an outcome of HYK (Dohoo and Martin, 1984; Duffield et al., 2009). However, in our study, we explored the role of milk yield on the association between HYK and reproductive performance. It is important to make clear that this study was observational and designed to be hypothesis-generating. Consequently, our intention with this study was to determine whether the interaction between milk production and HYK+ merits further study.

The incidence of HYK observed in this study was lower than the incidence levels reported by other studies (Kaufman et al., 2016; Weigel et al., 2017). This difference can be attributed to the different sampling schemes between studies. Recently, Tatone et al. (2016) highlighted that the abundance of protocols, sampling periods, diagnostic devices, and case definitions used in studies related to HYK have led to differences in the frequency of HYK reported in the literature.

In this study, we diagnosed HYK by measuring BHB concentrations in blood twice between 3 and 10 DIM. This sampling scheme was selected based on the fact that the peak of HYK cases occurs at 5 DIM and HYK has a disease length of 5 d (McArt et al., 2012). Although this strategy aimed to avoid false-negative results, cases with a shorter duration or that occurred after 10 d postpartum could have been missed and thus, not included in our analyses, resulting in a lower incidence. In addition, the frequency of HYK can vary depending on the time of sampling. According to Seely et al. (2021), the nadir of BHB concentration in plasma occurs at morning feed delivery, and peak BHB concentration occurs 4 h after that. In this study, we measured BHB concentration consistently at morning feed delivery, providing another possible reason for the lower incidence described.

One limitation of the study was our inability to determine how many animals lacked daily milk yield records within the first week postpartum. Lack of daily milk yield records could have arisen as a consequence of the inability of the milking system to read electronic identification devices in the 3 farms with direct milk measurements. Although missing data can be a source of selection bias, we do not expect that the proportion of missing data is different from what we would observe on any commercial farm on which an electronic identification system was used. Moreover, we consider that the missing data were missing at random, given that the reason for the lack of records was not related to the exposure or the outcome in the study. This assumption was further supported by the similar distribution of cows across percentiles of milk yield in wk 1 (Table 1). Nonetheless, the estimates need to be interpreted with caution.

When we evaluated only the association between HYK and reproductive parameters without wk 1 milk yield stratification, we observed an effect on reproduction, especially when considering the pregnancy by 150 DIM and CCI outcomes. These results agree with previous reports that found evidence of an association (Walsh et al., 2007; Ospina et al., 2010) but conflict with results of other studies (Chapinal et al., 2012; McArt et al., 2012). Based on our results and considering that cows need to be in positive energy balance to fully express estrus behavior and become pregnant (Rutherford et al., 2016), we suspect that this discrepancy can be related to a difference in energy balance during the transition period. When we stratified our data set by early milk yield, we observed 2 notable findings that lend credence to the importance of energy balance in the relationship between HYK and reproductive success. First, cows with high and moderate wk 1 milk yield had higher reproductive performance compared with low-yielding cows, independent of their HYK status. Second, HYK was associated with reduced reproductive performance of low-yielding cows. These results suggest a trend of an effect modification caused by wk 1 milk yield that warrants further confirmatory evaluation. In addition, our results contradict the general perception that cows with high milk yield have low reproductive efficiency, a belief that has been previously challenged (LeBlanc, 2010; Bello et al., 2012). Instead, the low reproductive efficiency may be attributable to other causes, such as greater severity of postpartum negative nutrient balance or to an increased incidence of disease in early lactation (Gröhn and Rajala-Schultz, 2000; Lucy, 2001; Drackley and Cardoso, 2014). Consequently, we presume that HYK+ cows that maintained a milk yield level in early lactation over the 25th percentile adapted better to their new physiological state by sustaining adequate nutrient balance and avoiding metabolic imbalance and diseases, and are thus more likely to have successful reproductive performance.

A strength of this study was the enrollment of cows in multiple herds varying in sizes and management strategies representing a variety of the dairy herds in the Upper Midwest region of the United States. Milk yield recording methods also varied among farms. The benefits of the inclusion of farms in which milk yield was estimated by DC305 instead of based on daily milk weights are the improvement of external validity and the increase in precision of the estimates by increasing the study sample size. However, this was also considered a limitation of this study because we had no information on the performance of the milk yield estimation equation used in the 2 farms without daily milk weight measurements. Moreover, because there was potential for the inclusion of farms that used estimation equations for milk yield in our analyses to bias the study results, we conducted a sensitivity analysis, finding that the inclusion of these farms resulted in a bias, albeit relatively low, on the outcomes (10 to 13%), warranting acknowledgment as a possible limitation. Last, due to our observational sampling strategy, HYK and wk 1 milk yield happen at the same time after calving, and thus the temporality of these variables is not clear. We argue that both variables begin during transition with increased fat mobilization. However, caution should be taken when interpreting the results of this exploratory study.

In conclusion, our results suggest that early lactation milk yield plays a role in the association of HYK with reproductive performance. Among low-yielding cows, HYK+ cows were less likely to become pregnant. Conversely, reproductive performance was similar among mid- and high-yielding cows, independent of HYK status, between 3 and 10 DIM. The results suggest that it could be beneficial to consider milk yield in addition to the measurement of BHB concentration in blood when monitoring dairy cows in early lactation to better differentiate between HYK cows that will and will not have negative downstream outcomes as a consequence of HYK. This hypothesis warrants further investigation in a larger confirmatory study, evaluating early lactation milk yield as a potential effect modifier of the association of HYK with reproductive performance as well as with other major health and productive outcomes.
